# When the microbiome helps the brain‐current evidence

**DOI:** 10.1111/cns.14076

**Published:** 2023-01-04

**Authors:** Jovana Drljača, Nataša Milošević, Maja Milanović, Ludovico Abenavoli, Nataša Milić

**Affiliations:** ^1^ Faculty of Medicine, Department of Pharmacy University of Novi Sad Novi Sad Serbia; ^2^ Department of Health Sciences University Magna Graecia Campus “Salvatore Venuta” Catanzaro Italy

**Keywords:** alzheimer disease, gut microbiota, parkinson disease, psychiatric disease, psychobiotics

## Abstract

The gut microbiota‐brain axis has been recognized as a network of connections that provides communication between the gut microflora and both central and autonomic nervous system. The gut microbiota alteration has been targeted for therapy in various neurodegenerative and psychiatric disbalances. Psychobiotics are probiotics that contribute beneficially to the brain function and the host mental health as a result of an interaction with the commensal gut bacteria, although their mechanism of action has not been completely revealed. In this state‐of‐art review, the findings about the potential therapeutic effects of the psychobiotics alone or in combination with conventional medicine in the treatment of neurodegenerative diseases such as Alzheimer's disease and Parkinson's disease, as well as in some psychiatric diseases like depression, schizophrenia, and bipolar disorder, have been summarized. The evidence of the psychobiotics therapeutic outcomes obtained in preclinical and clinical trials have been given respectively for the observed neurodegenerative and psychiatric disorders.

## Graphical abstract

Gut microbiota communicates with the brain in a bidirectionnal manner via n.vagus, microbial metabolites such as short chain fatty acids, cytokines, and neurochemicals.Gut microbiota dysbiosis has been implicated in pathophysiology of neurodegenerative diseases such as Alzheimer’s and Parkinson’s diseases, as well as of the psychiatric disorders like depression, schizophrenia and bipolar disorder.

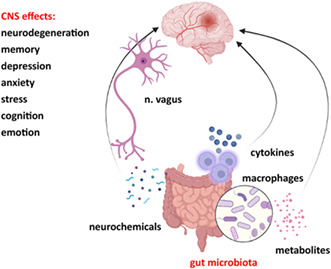

## 1 INTRODUCTION

The probiotics usage has been known much earlier than microbes were discovered. Fermented dairy products were painted on Egyptian hieroglyphs, and Tibetan peasants traditionally used fermented yak milk to preserve it during long journeys. In the early 19th century, scientists noticed the apparent health effects of fermented dairy products. Although Pasteur identified the responsible bacteria and yeasts for the fermentation process, no health effects had been attributed to the microbes.^1^ In 1908, Metchnikoff associated the Bulgarians' long life span with the *Lactobacillus* species from regularly consuming fermented milk and their presence in the gut.^2^ Tissier isolated *Bifidobacterium* species in infants and claimed that they could displace gut pathogens.^3^ These findings have catalyzed research into health‐promoting microbes and their role in disease prevention. In one of the earliest human studies, in 1922, *Lactobacillus acidophilus* was used in 30 patients with chronic constipation, diarrhea, and eczema, showing improvement in all three conditions.^4^ Soon after, the beneficial effects of *Lactobacillus acidophilus* were confirmed in patients with constipation and mental illness.^5^ Again, scientists brought to the light bidirectional communication between brain and gut, claiming that emotional state could modify gut function. Moreover, Canon^6^ showed that certain parts of the gastrointestinal tract, as well as the urinary bladder, were susceptible to mental state.

In the 21st century, the growing scientific research highlighted the bond between the brain and the gut.^7^, ^8^ Alterations in the gastrointestinal microbiome, known as dysbiosis, have been observed during the onset and development of mental disorders. Numerous adverse psychiatric reactions to antibiotics have been reported, even in patients with no history of psychiatric illness.^9^ To manage depression, Logan and Katzman (2005)^10^ proposed the probiotics as concomitant therapy with antidepressants. Many studies were done to shed light on the interplay between the gut microbiome and the brain, and thus, the digestive system‐brain axis concept was created.^11^ Preclinical and clinical studies suggested that administration of beneficial microbes may reduce depression, anxiety, stress, neuroinflammation, neurodegeneration, eliminate panic attacks, hypochondriac behavior and somatization, and improve cognition.^12^ Furthermore, early‐life perturbations in the gastrointestinal microbiome influenced neurodevelopment, with emerging mental health issues years later.^13^

Dinan et al. (2013)^14^ coined the term psychobiotics, as probiotics, which, if taken in appropriate amounts, have positive mental health effects. Magnetic resonance spectroscopy revealed that the oral administration of *Lactobacillus* leads to increased levels of γ‐aminobutyric acid (GABA), N‐acetylaspartate, and glutamate in the brain, assuming that these neuroactive molecules were enrolled in the mechanism of *Lactobacillus* strain action. Sarkar et al. (2016)^15^ suggested that the definition of psychobiotics could be expanded to any substance that confer positive alterations in the microbiome. The prebiotics could also be part of the psychobiotics, supporting the psychobiotic bacteria growth and thus contributing mental health. Afterward, similar terminology has emerged: paraprobiotics, also known as inactivated probiotics, and postbiotics, which are non‐viable bacterial cells, their products, or metabolites of live probiotic microorganisms. Both of them showed physiological benefits to the host.^16^ Recently, it has been suggested that the concept of postbiotics should be expanded to comprise paraprobiotics in the definition.^17^ Although many details in the mechanism of action remain unraveled, today, it is believed that the intestinal microbiome affects the brain and human behavior.

## 2 GUT MICROBIOTA‐BRAIN AXIS

A key step in understanding the psychobiotics mechanism lies in studying the ongoing communication between the gut microbiota and the brain.^15^ In humans, substantial evidence of this interaction happened more than 25 years ago. Patients suffering from hepatic encephalopathy showed the dramatic improvements after oral antibiotics.^18^ The intestinal flora interacts closely in a bidirectional manner, linking to the intestine permeability, immune system, and entero‐endocrine signaling with the cognitive and emotional brain centers.^19^ The gut microbiota‐brain axis binds the neuroendocrine and neuroimmune systems, as well as the autonomic nervous system with the intestinal microbiota.^19^, ^20^, ^21^

**FIGURE 1:**
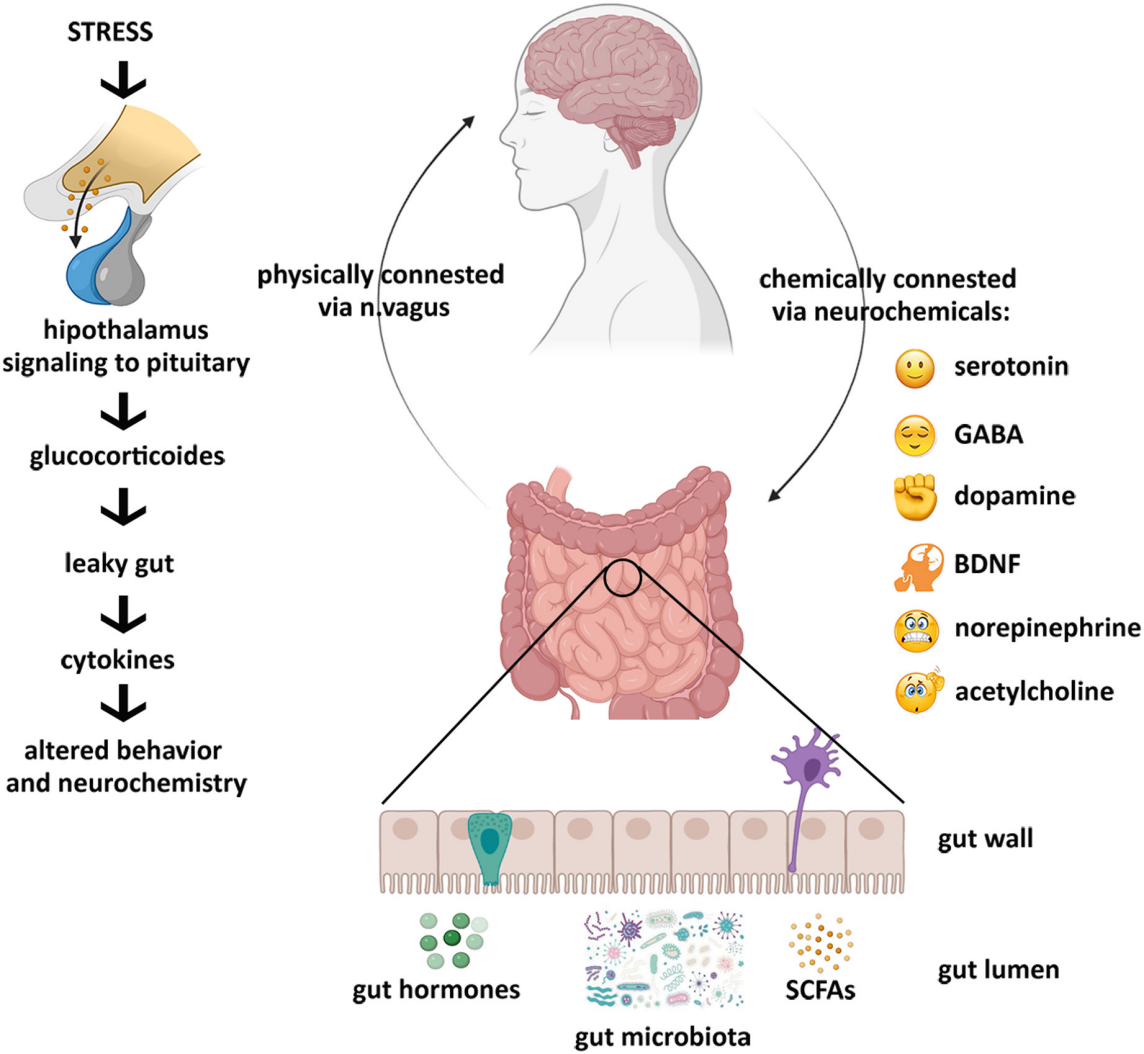
Gut microbiota–brain axis. Brain acts on the gut microbiome through the HPA axis and vagus nerve. In turn, gut dysbiosis acts on the brain through the vagus mechanism and immune endocrine pathway to cause neuronal degeneration and behavioral abnormalities.

The hypothalamic–pituitary–adrenal axis (HPA) is considered to be the main part of the neuroendocrine system that provides an adequate hormonal response to the stress.^22^ The stress hormones, glucocorticoids, disrupt gut barrier integrity through alterations in tight junctions, leading to a leaky gut and triggering inflammatory immune response (Figure 1).^23^

It is recognized that early colonization of the intestinal microbiota affects certain aspects of brain function and behavior, including neuroendocrine responses to stress. Sudo et al. (2004)^24^ showed that germ‐free mice exerted enhanced physiological reactions to stress in comparison to control. However, following the gut microbiota recolonization with probiotics, these abnormal reactions were reversible. The manifestation of homeostatic effects of probiotics on neuroendocrine physiology is highly significant, and it indicates new therapeutic possibilities.^25^

The development of the intestinal immune system largely depends on exposure to microorganisms.^26^ Gut microbiome alteration is associated with aberrant immune response due to the overproduction of cytokines through the HPA axis modulation.^27^ The elevation of circulating pro‐inflammatory cytokines IL‐6 and TNF‐α, with concomitant activation of microglia, brain resident macrophages, is involved in induced depressive and anxiety states, and other affective disorders.^28^, ^29^ Nonetheless, certain probiotics can reverse the microglia activation and decrease the levels of pro‐inflammatory cytokines, mediating anti‐inflammatory response.^15^

The vagus nerve is pivotal in coordinating parasympathetic activity with afferent terminals under the intestinal epithelium. Gut microbiota signals, transported to the brain, alter host behavior and possibly cause lethargy, loss of appetite, depression, or anxiety state.^30^ In several animal studies, it has been found that n. vagus mediated the interplay between psychobiotics and their psychophysiological effects, since vagotomy has eliminated the response to psychobiotic administration.^31^, ^32^, ^33^

The enteric nervous system (ENS) is responsible for coordinating the various digestive functions by forward and backward brain signal propagation, all via the vagus nerve.^34^ Myenteric neurons are close to the gut lumen, facilitating their contact with the microbiota.^8^ In addition, plenty of evidence suggested that gut bacteria modulated the ENS, regulating electrophysiological thresholds of myenteric neurons.^32^, ^35^, ^36^, ^37^

According to both preclinical and clinical evidences, changes in beneficial bacteria may have significant health consequences, while certain factors such as infection, drug use, diet, exercise, environment, social interactions, and stress can alter the microbiome.^12^, ^38^, ^39^ This determined changes in motility and intestinal secretion, caused visceral hypersensitivity, and lead to modification of the enteroendocrine and immune system.^19^

## 3 PSYCHOBIOTICS AND NEUROACTIVE MOLECULES

Gut microbiota produces a broad range of neuroactive molecules, signaling in the crosstalk between the gut microbiome and host metabolism (Figure 1). Due to the chemical and functional resemblance, these metabolites act as human neurotransmitters or neuromodulators. Serotonin (5‐HT), dopamine (DA), noradrenaline (NA), GABA, acetylcholine (Ach), as well as short‐chain fatty acids (SCFAs), produced by the gut microbiota via the metabolism of indigestible fibers, are of special interest.^40^, ^41^, ^42^, ^43^

Altered levels of 5‐HT and DA are implicated in several mental health and neurological diseases.^8^, ^44^ NA is able to modulate cognitive functions, learning, memory processes, and mood disturbances.^45^ GABA and Ach are the main inhibitory/excitatory neurotransmitters. Muller et al. (2021)^46^ demonstrated that SCFAs composition is associated with psychiatric and gastrointestinal symptoms in adults with affective or anxiety disorders. Therefore, psychobiotics control the neural excitatory‐inhibitory balance and modulation of the host's response to anxiety and depression. Due to these effects of microbially produced neuroactive molecules, psychobiotics have been suggested as a promising alternative or supportive therapy in treating neuropsychiatric disorders. In Table 1 results of different psychobiotic treatments associated with neurodegenerative and psychiatric diseases are summarized.

**TABLE 1:** Summary of different psychobiotic treatment results in neurodegenerative and psychiatric diseases

Subjects	Treatment	Outcome	Authors, year
Alzheimer's disease			
3 × Tg‐AD^†^ mice	*Lactobacillus plantarum* PS128	↓ Gliosis Regulated the propionic acid levels Regulated glycogen synthase kinase 3 beta activity	Huang et al., 2021^47^
15–17 months old Lister Hooded rats	*Lactobacillus acidophilus* CUL60, *Lactobacillus acidophilus* CUL21, *Bifidobacterium bifidum* CUL20, and *Bifidobacterium lactis* CUL34	↑ GABA ↑ Glutamine ↑ BDNF^†^ ↓ Microglial activation Restored synaptic plasticity ↑ Task‐specific memory ↑ Spatial learning and memory	O'Hagan et al., 2017^48^
Aβ^†^‐injected ddY mice	*Bifidobacterium breve* A1	↓ Aβ‐induced gene expression ↑ Plasma acetate level ↑ Spatial memory Prevented Aβ^†^‐induced cognitive dysfunction	Kobayashi et al., 2017^49^
D‐galactose‐injected Wistar rats	*Lactobacillus plantarum* MTCC1325	↓ AchE^†^ ↑ Ach^†^ in the cortex and hippocampus ↓ Amyloid plaques in the cortex and hippocampus ↑ Spatial memory	Nimgapalle and Kuna, 2017^50^
3 × Tg‐AD^†^ mice	SLAB51 formulation: *Streptococcus thermophilus*, *Bifidobacterium*. *longum*, *Bifidobacterium breve*, *Bifidobacterium infantis*, *Lactobacillus acidophilus*, *Lactobacillus plantarum*, *Lactobacillus*. *paracasei*, *Lactobacillus delbrueckii* subsp. *bulgaricus*, *Lactobacillus brevis*	↑ Ghrelin ↑ Leptin ↑ GIP^†^ ↓ Aβ^†^ deposits ↓ Cathepsin B ↑ Cathepsin L	Bonfilli et al., 2017^51^
Aβ^†^‐injected Wistar rats	*Lactobacillus acidophilus*, *Lactobacillus fermentum*, *Bifidobacterium lactis*, and *Bifidobacterium longum*	↓ Amyloid plaques ↓ Inflammation ↓ ^MD†A†^ and SOD^†^ ↑ Spatial memory Changed fecal microbiota composition	Athari et al., 2018^52^
5XFAD Tg mice	*Lactobacillus plantarum* C29	↓ Amyloid plaques ↓ Caspase 3 expression ↓ NF‐κB activation ↑ BDNF^†^ ↑ Microglial activation	Lee et al., 2021^53^
AD^†^ patients	*Lactobacillus acidophilus, Lactobacillus casei*, *Bifidobacterium bifidum*, and *Lactobacillus fermentum*	↑ MMSE^†^ score ↓ MDA^†^ ↓ CRP^†^ ↓ Triglyceride	Akbari et al., 2016^54^
AD^†^ patients	*Lactobacillus fermentum*, *Lactobacillus plantarum*, *Bifidobacterium lactis*, *Lactobacillus acidophilus*, *Bifidobacterium bifidum*, and *Bifidobacterium longum*	↑ TYM^†^ score ↑ GSH^†^ No effect on antioxidant status	Agahi et al., 2018^55^
AD^†^ patients	*Lactobacillus casei* W56, *Lactococcus lactis* W19, *Lactobacillus acidophilus* W22, *Bifidobacterium lactis* W52, *Lactobacillus paracasei* W20, *Lactobacillus plantarum* W62, *Bifidobacterium lactis* W51, *Bifidobacterium bifidum* W23 and *Lactobacillus salivarius* W24	↑ Kynurenine ↑ Immune cell activation ↓ Fecal zonulin Changed stool microbiota composition	Leblhuber et al., 2018^56^
AD^†^ patients	*Lactobacillus acidophilus*, *Bifidobacterium bifidum*, *Bifidobacterium longum*, and selenium	↑ MMSE^†^ score ↓ CRP^†^ ↓ Triglyceride ↑ GSH^†^ ↑ TAC^†^	Tamtaji et al., 2019^57^
Parkinson's disease			
MitoPark PD^†^ mice	*Bifidobacterium bifidum, Bifidobacterium longum, Lactobacillus rhamnosus, Lactobacillus rhamnosus* GG, *Lactobacillus plantarum* LP28, and *Lactococcus lactis* subsp. *lactis*	Improved gait Improved beam balance Improved motor coordination ↓ Dopaminergic neuronal loss Preserved TH^†^‐positive cells in substantia nigra	Hsieh et al., 2020^58^
MPTP^†^‐induced PD^†^ in C57BL/6 mice	*Lactobacillus rhamnosus* GG, *Bifidobacterium animalis lactis*, and *Lactobacillus acidophilus*	↑ Butyrate production ↑ BDNF^†^ ↓ MAO‐B^†^ in the striatum ↑ Dopamine synthesis Preserved the nigral dopaminergic neurons	Srivastav et al., 2019^59^
6‐OHDA^†^‐induced PD^†^ in C57BL/6 mice	SLAB51 formulation	↑ BDNF^†^ ↑ PPARγ^†^ ↓ Neuronal loss ↑ Antioxidative effects	Castelli et al., 2020^60^
MPTP^†^‐induced PD^†^ in C57BL/6 mice	*Lactobacillus plantarum* CRL2130, *Streptococcus thermophilus* CRL807, and *Streptococcus thermophilus* CRL808	↑ Motor skills ↑ TH^†^‐positive cells ↑ IL10 ↓ IL6 ↓ TNFα	Perez Visnuk et al., 2020^61^
PD^†^ patients	*Lactobacillus acidophilus* and *Bifidobacterium infantis*	↓ Abdominal pain ↓ Constipation ↓ Bloating	Georgescu et al., 2016^62^
PD^†^ patients	*Enterococcus faecium*, *Lactobacillus acidophilus*, *Lactobacillus paracasei*, *Lactobacillus rhamnosus*, *Bifidobacterium longum*, *Bifidobacterium bifidum*, and *Lactobacillus reuteri*	↑ SBM^†^ Improved stool consistency ↓ Constipation	Tan et al., 2021^63^
PD^†^ patients	*Lactobacillus casei* Shirota	Improved stool consistency ↓ Bloating ↓ Abdominal pain ↓ Constipation	Cassani et al., 2011^64^
PD^†^ patients	*Lactobacillus acidophilus, Bifidobacterium bifidum*, *Lactobacillus reuteri*, and *Lactobacillus fermentum*	↓ IL1 ↓ IL8 ↓ TNFα ↑ TNFβ ↑ PPARγ^†^	Borzabadi et al., 2018^65^
PD^†^ patients	*Streptococcus salivarius* subsp. *thermophilus*, *Enterococcus faecium*, *Lactobacillus rhamnosus* GG, *Lactobacillus acidophilus*, *Lactobacillus plantarum*, *Lactobacillus paracasei*, *Lactobacillus delbrueckii* subsp *bulgaricus*, and *Bifidobacterium (breve and animalis* subsp. *lactis*	↑ CBM^†^ ↓ Constipation Improved stool consistency	Barichella et al., 2016^66^
Depression			
Mice exposed to chronic restraint stress	*Bifidobacterium adolescentis*	↓ IL‐1β ↓ TNF‐α ↓NF‐κB p65 ↓ Iba1 ↑ BDNF^†^ in the hippocampus ↑ *Lactobacillus* level in feces ↓ *Bacteroides* level in feces	Guo et al., 2019^67^
Corticosterone‐induced depression in mice	*Lactobacillus paracasei* PS23, live or heat‐killed	Live PS23: ↓ Abnormal behavior ↑ BDNF^†^ in the hippocampus ↑ Serotonin in hippocampus, prefrontal cortex and striatum Heat‐killed PS23: ↓ Abnormal behavior ↑ BDNF^†^ in the hippocampus ↑ Dopamine levels in hippocampus and prefrontal cortex.	Wei et al., 2019^68^
Healthy Swiss mice	*Lactobacillus plantarum* 286	Anti‐depressant‐like effects Anxiolytic‐like effects	Barros‐ Santos et al., 2020^69^
Wistar rats exposed to chronic unpredictable mild stress	*Lactobacillus rhamnosus* JB‐1	↑ GABA ↑ Glutamate ↑ GSH^†^ ↑ N‐acetylaspartate	Kochalska et al., 2020^70^
C57BL/6J mice exposed to chronic stress	*Bifidobacterium breve* CCFM1025	Changed gut microbiota composition ↑ SCFAs^†^ ↑ 5‐Hydroxytryptophan ↑ BDNF^†^	Tian et al., 2020^71^
Corticosterone‐induced depression in Sprague–Dawley rats	*Lactobacillus plantarum* DP189	Improved memory and spatial learning ↓ Anhedonia ↓ IL‐1β ↓ TNFα ↓ BAX ↑ Bcl‐2	Zhao et al., 2020^72^
Lipopolysaccharide‐induced anxiety in adolescent CD1 mice	BGOS^†^	Anxiolytic effect ↓ IL‐1β ↑ 5‐HT2A receptor expression	Savignac et al., 2016^73^
C57BL/6J mice exposed to chronic psychosocial stress	FOS^†^ + GOS^†^	↑ Cecal acetate and propionate level ↓ Cecal isobutyrate level ↑ BDNF^†^ expression in the hippocampus ↓ L‐tryptophan, ↓ Corticosterone ↓ Proinflammatory cytokines	Burokas et al., 2017^74^
Sprague–Dawley rats exposed to chronic unpredictable mild stress	FOS^†^	↓ Depression‐like behaviors Repaired intestinal epithelia damages Changed fecal microbial composition	Chi et al., 2020^75^
C57BL/6J mice exposed to subchronic and mild social defeat stress	Heat‐killed *Lactobacillus helveticus* strain MCC1848	↑ The interaction time in the social interaction test ↑ Sucrose preference ratio in the sucrose preference test Modulated gene expression in the nucleus accumbens	Maehata et al., 2019^76^
Healthy C57BL/6 mice	Heat‐killed *Lactobacillus fermentum* and *Lactobacillus delbrueckii*	Sedative behavior effect ↓ Corticosterone level Changed gut microbiota composition	Warda et al., 2019^77^
Healthy C57BL/6J mice	Heat‐killed *Enterococcus faecalis* strain EC‐12	↓ Anxiety‐like behavior ↓ Depressive‐like behavior	Kambe et al., 2020^78^
Adults with moderate mood swings	*Lactobacillus helveticus* R0052 and *Bifidobacterium longum* R0175	Non‐effective in treating low mood No effects on inflammation	Romijn et al., 2017^79^
Moderately stressed adults	*Lactobacillus plantarum* DR7	↓ Symptoms of stress anxiety ↓ Total psychological scores ↓ Cortisol ↓ IFNγ ↓ TGFα ↑ IL10 Enhanced the serotonin pathway Stabilized the dopamine pathway	Chong et al., 2019^80^
Moderately stressed adults	*Lactobacillus plantarum* P8	↓ Scores of stress ↓ Anxiety ↓ IFNγ ↓ TNFα Enhanced memory and cognitive traits	Lew et al., 2019^81^
Healthy adults	Heat‐killed *Lactobacillus paracasei* MCC1849	Improved resistance to common cold infections Maintained a desirable mood state	Murata et al., 2018^82^
Young adult students preparing for the national examination	Heat‐inactivated *Lactobacillus gasseri* CP2305	↓ Anxiety ↓ Sleep disturbance Changed gut microbiota composition	Nishida et al., 2019^83^
Patients with major depressive disorder	*Lactobacillus plantarum* 299v + SSRI	Improved cognitive performance ↓ Kynurenine	Rudzki et al., 2019^84^
Adult patients with moderate depression	*Lactobacillus casaei* + *Lactobacillus acidofilus* + *Lactobacillus bulgarigus* + *Lactobacillus rhamnosus* + *Bifidobacterium breve* + *Bifidobacterium longum* + *Streptococcus thermophilus* + FOS + fluoxetine	↓ Depressive clinical symptoms	Ghorbani et al., 2018^85^
Adult patients with mild‐to‐moderate depression	*Lactobacillus helveticus* Rosell® − 52 + *Bifidobacterium longum* Rosell® − 175 + SAMe^†^ + magnesium oxide + vitamin B6	↓ PHQ‐9^†^ and HAM‐D^†^ scores	Ullah et al., 2022^86^
Adult patients with major depressive disorder	Freeze‐dried *Bifidobacterium* *breve* CCFM1025	↓ Serum serotonin turnover ↓ HDRS‐24^†^ and MADRS^†^ scores	Tian et al., 2022^87^
Schizophrenia			
Sprague Dawley rats	BGOS^†^ + olanzapine	↓ Olanzapine‐induced weight gain ↑ Cognitive flexibility ↑ Acetate production	Kao et al., 2018^88^
C57BL/6 mice on a high‐fat diet	*Akkermansia muciniphilasub* (Akk I subtype, GP01 strain) + olanzapine	Did not suppress the olanzapine‐induced weight gain ↑ Locomotion ↓ Insulin, total cholesterol, triglycerides ↓ ALT^†^, AST^†^ ↓ Gluconeogenesis and insulin resistance ↓ IL‐6 and TNFα	Huang et al., 2021^89^
Sprague Dawley rats	FOS^†^ + GOS^†^	↑ NMDA^†^ receptor subunits in the hippocampus ↑ NR2A subunits in hippocampus ↑ NR1 and d‐serine in frontal cortex	Savignac et al., 2013^90^
BALB/c mice	*Lactobacillus rhamnosus* JB‐1	↑ GABA in hippocampus and prefrontal cortex	Janik et al., 2016^91^
C57BL/6J mice	FOS^†^ + GOS^†^	↑ GABA‐B1 receptor gene expression in the hippocampus ↑ GABA‐B2 receptor gene expression in the hippocampus	Burokas et al., 2017^92^
Sprague Dawley rats	BGOS^†^	↑ NMDA^†^ cortical receptor function Improved performance in a set‐shifting task	Gronier et al., 2018^93^
SCZ^†^ patients	*Lactobacillus rhamnosus* GG and *Bifidobacterium animalis* subsp. *lactis* Bb12	No effects on SCZ^†^ symptoms (PANSS^†^ score) ↑ BDNF^†^ Patients were less likely to develop severe bowel difficulties	Dickerson et al., 2014^94^
Patients with SCZ^†^ and schizoaffective disorder	*Lactobacillus rhamnosus* GG and *Bifidobacterium animalis* subsp. *lactis* Bb12	No effects on SCZ^†^ symptoms (PANSS^†^ score) ↑ BDNF^†^ ↑ Macrophage inflammatory protein 1β.	Tomasik et al., 2015^95^
SCZ^†^ patients	*Lactobacillus rhamnosus* GG and *Bifidobacterium animalis* subsp. *lactis* Bb12	No effects on SCZ^†^ symptoms (PANSS^†^ score) but greater effects were seen for positive symptoms rather than negative symptoms Improvement in positive symptoms and bowel movement in *Candida albicans* seronegative males	Severance et al., 2017^96^
Bipolar disorder			
BD^†^ patients	*Bifidobacterium bifidum*, *Bifidobacterrium lactis*, *Bifidobacterium longum*, and *Lactobacillus acidophilus*	↓ Mania ↓ Depression	Shahrbabaki et al., 2020^97^
BD^†^ patients	*Lactobacillus casei* W56, *Lactobacillus acidophilus* W22, *Lactobacillus paracasei* W20, *Bifidobacterium lactis* W51, *Lactobacillus salivarius* W24, *Lactococcus lactis* W19, *Bifidobacterium lactis* W52, *Lactobacillus plantarum* W62, *Bifidobacterium bifidum* W23	↑ Attention ↑ Psychomotor processing speed ↓ Mania	Reininghaus et al., 2020^98^

Abbreviations: AD, Alzheimer's disease; AchE, acetylcholine esterase; Ach, acetylcholine; ALT, alanine‐transaminase; AST, aspartate‐transaminase; Aβ, amyloid beta; BD, bipolar disorder; BDNF, brain‐derived neurotrophic factor; B‐GOS, Bimuno™ galacto‐oligosaccharide; CBM, complete bowel movements; CRP, C‐reactive peptide; FOS, fructo‐oligosaccharides; GIP, gastric inhibitory polypeptide; GOS, galacto‐oligosaccharides; GSH, glutathione; HAM‐D, Hamilton Depression Rating Scale; HDRS‐24, Hamilton Depression Rating scale‐24 Items; MADRS, Montgomery‐Asberg Depression Rating Scale; MAO‐B, monoamine oxidase B; MDA‐ malondialdehyde; MMSE, Mini‐Mental State Examination; MPTP, 1‐methyl‐4‐phenyl‐1,2,3,6,‐tetrahydropyridine; NMDA, N‐methyl–D‐aspartate; PANSS, Positive and Negative Syndrome Scale; PD, Parkinson's disease; PHQ‐9, Patient Health Questionnaire‐9; PPARγ, peroxisome proliferator‐activated receptor gamma; SAMe, S‐adenosyl‐L‐methionine disulfate p‐toluenesulfonate; SBM, spontaneous bowel movements; SCFAs, short‐chain fatty acids; ^†^SCZ, schizophrenia; SOD, superoxide‐dismutase; TAC, total antioxidant capacity; TH, tyrosine hydroxylase; TYM, Test Your Memory; 6‐OHDA, 6‐hydroxydopamine.

## 4 NEURODEGENERATIVE DISEASES TREATMENT WITH PSYCHOBIOTICS

### 4.1 Alzheimer's disease

AD is a chronic neurodegenerative disease with a progressive decline in cognitive and memory function. Recently, poor gut microbiota diversity in AD patients has gained an ongoing interest followed by finding an additional molecular pathogenesis for AD. Novel findings suggest that autoimmune and autoinflammatory mechanisms are engaged in AD.^99^ The data suggested that SCFAs, such as butyric, propionic, acetic acids, and microbial metabolites in colon reduced the AD neuropathological features and other neurodegenerative diseases by providing alternative energy sources to the brain.^100^ Moreover, selected SCFAs may modulate neuroinflammation, a significant pathomechanism of the early and preclinical course of AD. Amyloid β abnormality, tau phosphorylation, neurotransmitter dysregulation, and oxidative stress in AD followed the derangement in the gut microbiota composition.^47^, ^101^ In addition, several probiotic strains, such as *Lactobacillus plantarum* and *Bifidobacterium infantis*, enhance gut barrier function via upregulation of tight junction expression and production of SCFAs.^102^ Bearing in mind the richness of the gut milieu with endotoxins and amyloid β, the maintenance of secure gut barrier is requisite to avoid inflammation.

#### 4.1.1 Preclinical probiotic supplementation in AD

Research on rodents showed that memory storage and cognition began to decline with age and were severely damaged in AD.^103^ Germ‐free mice displayed a decreased level of tight junction protein and decreased level of brain‐derived neurotrophic factor (BDNF) and N‐methyl‐D‐aspartate (NMDA) receptor expression in the cortex and hippocampus.^104^ BDNF and NMDA are proven to play a pivotal role in neuroplasticity, which loss is a significant indicator of the AD etiology.^105^ GABA and glutamine levels, brain metabolites, are enhanced by long‐term administration of *Lactobacillus* spp. and *Bifidobacterium* spp. to aging rats, improving task‐specific memory.^48^ Moreover, Li et al. (2020)^106^ noticed a correlation between the gut microbiota changes and increased amyloid β deposition in mice through stimulation of the MAPK signaling pathway.

Attempts have been made to untangle the effects of different probiotic strains on AD. Kobayashi et al. (2017)^49^ observed the anti‐inflammatory effects of *Bifidobacterium breve A1* strain and amelioration of cognitive dysfunction in AD mice. Nimgampalle and Kuna (2017)^50^ showed that *Lactobacillus plantarum* reduced amyloid β load and increased the acetylcholine level in the cortex and hippocampus, with improved spatial memory in AD mice. In addition, Bonfilli et al (2017)^51^ reported that SLAB51 probiotic formulation (*Bifidobacteria* and *Lactobacilli* mixture) decreased amyloid β aggregations and positively influenced on inflammatory cytokines levels, preventing the onset and delaying the AD progression in early‐stage AD. These probiotic strains were found to restore synaptic plasticity, followed by a decrease in microglial activation and increased BDNF, improving cognitive function and spatial learning.^48^ Another study found that the *L*.*acidophilus*, *L*.*fermentum*, *B*.*lactis*, and *B*. *longum* mixture attenuated the learning deficits and oxidative stress and improved spatial memory.^52^ *L*. *plantarum* was also shown to regulate microglia activation, reduced amyloid β load via suppressing NF‐κB activation in AD mice.^53^ These findings indicated a possible role of specific probiotic strains in enhancing memory and cognitive function.

#### 4.1.2 Clinical probiotic supplementation in AD

The *Firmicutes*/*Bacteroidetes* ratio has emerged as an indicator of intestinal microbiota health. It is shown that AD patients have decreased *Firmicutes* level along with increased *Bacteroidetes* level.^107^ Eskelinen et al. (2009)^108^ reported (21‐year follow‐up study of 1409 volunteers, aged 65–79) that the 3–5 cups of coffee per day in the middle ages reduced the risk of developing AD. The neuroprotective effect of coffee could be attributed to the antioxidant activity of polyphenols and the coffee seed fibers effected increased *Firmicutes*/*Bacteroidetes* ratio, bringing to reduced inflammation.^109^ However, the beneficial outcome of probiotics in AD patients is still lack of evidence because only 4 clinical trials based on *Lactobacillus* and *Bifidobacterium* lasting for maximum 12 weeks have been conducted. The results indicated that probiotics played a significant role in mitigating AD‐like symptoms in a multi‐targeted approach enhancing cognitive function.^54^, ^55^, ^56^, ^57^ In spite of current supportive evidence on health benefits, large‐scale, long‐term, randomized clinical trials are needed to posit a curative role of probiotics in AD patients.

### 4.2 Parkinson's disease

Parkinson's disease (PD) is a neurodegenerative disorder associated with the progressive loss of dopaminergic neurons in *substantia nigra* and abnormal intracellular aggregation of α‐synuclein.^110^ Motor impairment and characteristic brain pathology do not appear as symptoms until a fairly advanced stage of the disease. On the other hand, intestinal dysfunction, such as bloating, delayed gastric emptying, constipation, prolonged intestinal transit time with incomplete defecation, occurs years before the initiation of motor impairment.^111^, ^112^

Dysbiosis in gut microbiota has also been reported in PD. Decrease of *Prevotellaceae* in stool samples of PD patients,^113^ along with increased *Lactobacilliceae*, are found to be associated with lower ghrelin level, a gut hormone that maintains normal dopamine function.^114^ A study observed an antiinflammatory bacteria depletion, such as genus *Blautia*, *Roseburia*, and *Coprococcus* in stool samples of PD patients, along with a reduction in *Lactococcus* bacteria.^115^ It is assumed that this shift from predominantly antiinflammatory phenotype toward the proinflammatory phenotype of the gut microbiota contributes the increased gut permeability and decreased dopamine function.^116^ Furthermore, a significantly reduced abundance of SCFA (butyrate, acetate, propionate)‐producing bacteria was found in PD patients.^117^

#### 4.2.1 Preclinical probiotic supplementation in PD

PD mice supplemented daily with a six probiotic strains mixture (*B*. *bifidum*, *B*. *longum*, *L*. *rhamnosus*, *L*. *rhamnosus GG*, *L*. *plantarum*, and *L*. *lactis*) for 16 weeks showed better balance, gait, and coordination which persisted for 8 weeks. Moreover, a reduced dopaminergic neuronal degeneration was observed.^58^

Neuroprotective probiotics effect was also reported in rotenone‐ and 1‐methyl‐4‐phenyl‐1,2,3,6‐tetrahydropyridine (MPTP)‐induced PD mice model. *L*. *rhamnosus GG*, *B*. *animalis lactis*, and *L*. *acidophilus* mixture enhanced the butyrate production and elevated the BDNF level, which are associated with rescuing dopaminergic neurons from toxicity and promoting cell survival and proliferation. Furthermore, an inhibition of monoamine oxidase B contributed to the dopamine synthesis and survival of dopaminergic neurons.^59^ In accordance, the study conducted on a 6‐hydroxydopamine‐induced PD mouse model with a nine probiotic strains cocktail named SLAB51 showed that the reduced neuronal loss was mediated through the BDNF upregulation. Also, the peroxisome proliferator‐activated receptor gamma (PPAR‐γ) signaling pathway was activated, with ameliorating antiinflammatory and antioxidative effects.,^60^ Perez Visnuk et al. (2020)^61^ reported proinflammatory cytokines IL‐6 and TNF‐α reduction, with an antiinflammatory cytokine IL‐10 increment in PD mice treated with probiotics.

#### 4.2.2 Clinical probiotic supplementation in PD

Several clinical studies reported beneficial probiotics effects in PD patients, especially in combating constipation, a common symptom in PD with a prevalence of up to 70%.^62^, ^63^, ^118^ The first clinical trial conducted in 2011 highlighted that fermented milk with *Lactobacillus casei* administration improved stool consistency, defecation, decreased bloating, and abdominal pain in PD patients suffering from constipation.^64^ Delayed gastric emptying, also common in PD patients, was found to be accelerated after the *Lactobacillus reuteri* administration.^119^ In the randomized, double‐blind, placebo‐controlled clinical study, *L*. *acidophilus*, *L*. *fermentum*, and *B*. *bifidum* supplementation for 12 weeks reduced the expression of proinflammatory cytokines (IL‐1, TNF‐α) and oxidative markers, and increased the antiinflammatory factors expression (TGF‐β, PPAR‐γ).^65^ According to Barichella et al. (2016),^66^ the number of spontaneous and total bowel movements were improved in PD patients treated with probiotics for 4 weeks.

## 5 POSSIBLE PSYCHOBIOTIC TREATMENT IN THE MOST COMMON PSYCHIATRIC DISEASES

### 5.1 Depression

Although the depression is a complex chronic mood disorder associated with various etiology factors, there is a growing scientific evidence that the intestinal microbiota derangement is implicated in its pathophysiology.^120^ Luo et al. (2018)^121^ showed that changes in the gut microbiota composition altered mice behavior toward anxiety, depression, and even autism. The fecal microbiota transplantation performed from depression suffering patients into rats with entire microbiota previously removed, resulted in depression‐like behavior of the animals, suggesting that the transplantation of the abnormal microbiome could lead to the depression “transmission.”^122^ Clinical studies indicated that the gut microbiota of depressed patients differs significantly from healthy subjects.^8^ Antibiotics can also destroy important intestinal microorganisms and thus induce digestive‐brain dysfunction, which may cause an increased incidence of various diseases, including mental disorders like depression.^123^

The signal dysfunction of GABA, a major inhibitory neurotransmitter in the CNS, is associated with anxiety and depression. *Lactobacillus* and *Bifidobacterium* are capable of metabolizing glutamate to GABA. An *in vivo* experiment with *L*. *rhamnosus* reported alterations in GABA receptor expression in brain regions related to stress.^8^ In addition, pathogens and their metabolites may induce brain inflammation through circulation and cytokine cascade reactions, which further affect the various brain processes involved in mood and behavior.^124^ Pro/prebiotics exerted the beneficial effects as adjuvant therapy in mood disorders through the regulation of inflammatory markers, neurotransmission of serotonin, GABA, and BDNF and reducing HPA activity.^14^, ^125^

#### 5.1.1 Preclinical probiotic supplementation in depression

The psychobiotics positive effects in depressive disorders are studied mostly on three rodent models, such as the corticosterone‐induced depression model, chronic unpredictable mild stress, and chronic restraint stress model. The most tested probiotics were *L*. *plantarum*, *L*. *casei*, *L*. *rhamnosus*, *B*. *breve*, *B*. *infantis*, and *B*. *adolescentis*.^67^, ^68^, ^69^, ^70^, ^71^, ^72^ These studies showed attenuation of proinflammatory cytokines (IL‐1β and TNF‐α), apoptosis (downregulation of *BAX* with upregulation of *Bcl‐2*), and the tryptophan increase (a serotonergic precursor), BDNF, and GABA, along with mitigation of anxiety‐ and depression‐like behavior.

The prebiotics effects on mood disorders have been examined to a much less extent than probiotics. Nevertheless, studies done on fructooligosaccharides (FOS) and galactooligosaccharides (GOS) corroborated the psychobiotics effects regarding the alteration of behavior and neurochemistry. FOS and GOS supplementation, alone or in combination, yielded antidepressant outcomes.^73^, ^74^, ^75^ Supplementation with postbiotics, heat‐killed *L*. *helveticus*,^76^ *L*. *fermentum*, and *L*. *delbrueckii*,^77^ or heat‐killed *Enteroccocus fecalis* ^78^ subtly but distinctly changed the gut microbiota composition, reduced the baseline corticosterone level, and decreased anxiety‐ and depression‐like behavior.

#### 5.1.2 Clinical probiotic supplementation in depression

The clinical trials’ results regarding the effects of different probiotic strains in mitigating depressive‐like behavior are inconsistent. Three clinical trials demonstrated that the probiotic supplementation did not reverse behavioral deficits in patients with depression.^79^, ^80^, ^81^ In contrast, several studies showed beneficial neurobehavioral effects of probiotics and prebiotics in combination.^126^, ^127^ Moreover, heat‐killed *Lactobacillus paracasei* were efficient in maintaining the desirable mood state in healthy adults.^82^ Heat‐killed *Lactobacillus gasseri* reduced anxiety and altered the gut microbiota composition in young adult students preparing for the national examination.^83^

In concomitant therapy with antidepressants, probiotics exerted improvement in overall mood.^84^ Ghorbani et al. (2018)^85^ reported the improved depression clinical symptoms when specific probiotics and FOS were applied as an adjuvant therapy to the antidepressant drug fluoxetine.

### 5.2 Schizophrenia

Schizophrenia is a debilitating psychiatric disorder, with symptoms characterized as positive (aberrant flow of thoughts, delusions, hallucinations), and negative (social withdrawal, lack of motivation, apathy).^128^ Recently, dysbiosis has also been noticed in schizophrenic patients. Shen et al. (2018)^129^ and Zhang et al. (2020)^130^ found reduced *Roseburia* and *Faecalibacterium* levels in stool samples in schizophrenia patients. Noteworthy, both genera produce butyrate, maintaining the intestinal barrier secure.^131^, ^132^ Schizophrenia incidence has been found to correlate with the *Clostridium difficile* gut increase due to the phenylalanine derivatives production which controls catecholamine levels. Catecholamines, especially dopamine, are notably elevated in schizophrenia.^133^, ^134^ Interestingly, a significant *Lactobacilli* elevation in schizophrenia patients was noticed, which even correlated with the severity of symptoms.^135^

#### 5.2.1 Preclinical probiotic supplementation in schizophrenia

In female rats with olanzapine therapy, an increase in *Firmicutes* and a decrease in *Bacteroidetes*, with elevated systemic inflammatory markers IL‐6, IL‐8, TNF‐α, and IL‐1β were reported.^136^ In addition, several studies observed that prebiotics and probiotics could alleviate the side effects caused by antipsychotic therapy. Kao et al. (2018)^88^ and Huang et al. (2021)^89^ showed that the use of prebiotics and probiotics, respectively, as adjuvant therapy to olanzapine attenuated weight and metabolic disturbances in female animals.

Prebiotics (FOS and GOS) or certain probiotic strains (*Lactobacillus rhamnosus* and *Bifidobacterium infantis*) administration in animal models was found to elevate hippocampal levels of BDNF, GABA, and NMDA receptor gene expression.^90^, ^91^, ^92^, ^93^ These changes are relevant to schizophrenia, given that NMDA and GABA hypofunction in signaling, and decreased level of BDNF are thought to contribute to cognitive decline and psychotic symptoms.^137^

#### 5.2.2 Clinical probiotic supplementation in schizophrenia

Dickerson et al. (2014)^94^ investigated the effects of a *Bifidobacterium lactis* and *Lactobacillus rhamnosus* formulation, as adjuvant antipsychotic treatment. The probiotics failed to impact positive and negative manifestations, however, they elevated the BDNF level and improved gastrointestinal symptoms.^95^ The beneficial probiotics usage was observed in seronegative male schizophrenia patients, decreasing *Candida albicans* IgG serum levels.^96^ Flowers et al. (2019)^138^ found that the prebiotic raw unmodified potato starch supplementation in patients with antipsychotic therapy increased the *Actinobacteria* abundance. As patients with schizophrenia often suffer from enhanced stress response, compromised nutritional status, enhanced inflammatory status, and constipation, probiotics have promising therapeutic potential, alone or in combination with antipsychotics.^139^

### 5.3 Bipolar disorder

Bipolar disorder is severe neuropsychiatric disease, characterized by extreme mood swings that include emotional highs (mania) and lows (depression) and may also have gut dysbiosis etiology.^140^ Bipolar mania is twice as likely in patients who have been recently treated with antibiotics than in other patients.^141^ *Actinobacteria* and *Corinobacteria* increased levels, and *Faecalibacterium* and *Ruminococcaceae* decreased levels were reported in bipolar patients' stool samples, which correlated with the severity of symptoms.^142^, ^143^ Although Aizawa et al. (2019)^144^ reported no difference in the amount of *Lactobacillus* and *Bifidobacterium* stool samples of bipolar disorder patients comparing to healthy controls, a negative correlation was found in the *Lactobacillus* level and sleep, and the *Bifidobacterium* level and cortisol.

The HPA axis dysregulation, chronic inflammation, and abnormal monoamine function could cause bipolar disorder.^145^, ^146^, ^147^, ^148^ Hence, psychobiotic supplementation could be useful through the modulation of the aforementioned bipolar disorder causes and may help in treating microbiome dysbiosis and increased intestinal permeability. Also, given the high prevalence of gastrointestinal symptoms (diarrhea, satiety) in this patients, psychobiotic supplementation could form a potential add‐on therapy.

#### 5.3.1 Clinical probiotic supplementation in bipolar disorder

Akkasheh et al. (2016)^149^ reported that probiotics containing *Bifidobacterium bifidum*, *Lactobacillus casei*, and *Lactobacillus acidophilus* significantly reduced depressive symptoms. In line with this, Shahrbabaki et al. (2020)^97^ indicated that probiotic consumption alleviated the severity of mania and depression over time. Significant improvements to attention and psychomotor processing speed were achieved after 1 and 3 months of probiotics treatment, respectively, indicating the potential beneficial effects of probiotics in improving cognitive function.^98^ The lower rehospitalization rate was reported after probiotic supplementation as add‐on therapy in bipolar disorder patients .^150^

## 6 CONCLUSION

“Let food be thy medicine and medicine be thy food” is one of the antient quote that is so important nowadays.^151^ Recent studies have justified it considering a modified version: “Let food for your microbes be medicine for your brain.”^11^ Abnormal microbiota is undoubtedly involved in the etiology and pathophysiology of neurodegenerative disorders, behavioral and mood alterations, and it is likely to be the target of future therapies. However, in line with differences in food preferences, environment, and lifestyle, an outstanding issue is to define the normal gut microbiota, due to interindividual and geographic differences in gut microbiota composition.

Studying the whole ecosystem, gut microbiota and probiotics, is requisite in understanding the interplay between the gut and host health. The novel class of probiotics – psychobiotics – has shown to be helpful in improving CNS functions, such as neuronal degeneration, memory, depression, anxiety, and mood, modulating the HPA, inflammation, and neurochemical production. The usage of psychobiotics alone or in combination with conventional medicine could contribute to the development of new therapeutic strategies; however, more research is needed due to the scarcity of the clinical data.

Not all psychobiotics are probiotics, and *vice versa*, not all probiotics have psychobiotic potential. An increasing number of preclinical and clinical research reported these promising psychobiotics effects, with no side effects. At present, the discrepancies between the results from laboratories occur due to the rapid microbiome testing commercialization leading to the misinterpretation of the results and the lack of consensus in general. Therefore, multi‐omics approach would enable comprehensive insight into the linkage between the gut microbiota composition and related diseases, as a promising tool for further research.

## CONFLICT OF INTEREST

The authors declare they have no competing interests.

## Data Availability

The data that support the findings of this study are available from the corresponding author upon reasonable request.
